# Nilotinib Is More Potent than Imatinib for Treating Plexiform Neurofibroma *In Vitro* and *In Vivo*

**DOI:** 10.1371/journal.pone.0107760

**Published:** 2014-10-23

**Authors:** Jiang Wei, Marcus Freytag, Yvonne Schober, Wolfgang A. Nockher, Victor F. Mautner, Reinhard E. Friedrich, Paul W. Manley, Lan Kluwe, Andreas Kurtz

**Affiliations:** 1 Laboratory for Tumor Genetics, Department of Neurology, University Medical Center Hamburg-Eppendorf, Hamburg, Germany; 2 Department of Maxillofacial Surgery, University Medical Center Hamburg-Eppendorf, Hamburg, Germany; 3 Institute of Laboratory Medicine and Pathobiochemistry, Philipps University Marburg, Marburg, Germany; 4 Novartis Institute of Biomedical Research, Basel, Switzerland; 5 Berlin Brandenburg Center for Regenerative Medicine, Charité-Universitätsmedizin Berlin, Berlin, Germany; 6 Seoul National University, College of Veterinary Medicine and Research Institute for Veterinary Science, Seoul, Republic of Korea; Virginia Commonwealth University, United States of America

## Abstract

Plexiform neurofibromas (PNFs) are benign nerve sheath tumors mostly associated with neurofibromatosis type 1. They often extend through multiple layers of tissue and therefore cannot be treated satisfactorily by surgery. Nilotinib is a tyrosine kinase inhibitor used to treat leukemia, with advantages over the prototype imatinib in terms of potency and selectivity towards BCR-ABL, and the DDR, PDGFR, and KIT receptor kinases. In this study, we compared efficacies of the two drugs on cultured cells of PNF *in vitro* and on xenografted tumor fragments on sciatic nerve of athymic nude mice. Xenografts were monitored weekly using a high resolution ultrasound measurement. Treatment with nilotinib at a daily dose of 100 mg/kg for four weeks led to a reduction of the graft sizes_std_ by 68_±7_% in the 8 treated mice, significantly more than the 33_±8_% reduction in the 8 untreated mice (P<0.05) and the 47_±15_% in the 7 mice treated with imatinib (P<0.05). The peak plasma nilotinib concentration 6.6_±1.1_ µM is within the pharmacological range of clinical application. Imatinib, but not nilotinib significantly hindered body weight increase of the mice and elevated cytotoxicity of mouse spleen cells (P<0.05). Our results suggest that nilotinib may be more potent than imatinib for treating PNFs and may also be better tolerated. Imatinib seems to have some off-target effect in elevating immunity.

## Introduction

Plexiform neurofibromas (PNFs) are benign tumors originating from peripheral nerve sheath and mostly associated with neurofibromatosis type 1 (NF1), a tumor suppressor gene syndrome [1]. Depending on their location, size and growth type, PNFs can cause pain and disfigurement, functional impairment of vision, mobility, bladder and bowel [2]. PNFs have a high risk of malignant transformation into malignant peripheral nerve sheath tumors (MPNST) which is the leading cause of NF1-related death [3]–[5]. The lifetime risk of MPNST for NF1-patients has been estimated to be about 8 to 13% and thus is more than 1000 times higher for these patients than for the general population. The lifetime risk to develop an MPNST increases to 30–50% in patients with NF1 and PNF [6]–[8]. Since PNFs often extend through multiple layers of tissue, total resection is usually not possible without damaging functions and organs [9]–[10].

Nilotinib (Tasigna; Novartis Pharmaceuticals) is an orally active tyrosine kinase inhibitor which targets ABL (and the oncogenic BCR-ABL), together with several receptor tyrosine kinases including those for stem cell factor (c-KIT), collagen (DDR-1/-2) and platelet-derived growth factor (PDGFR-α/-β) [11]–[13]. Nilotinib has a number of advantages over imatinib (Glivec; Novartis Pharmaceuticals), including a different toxicity profile and a lower incidence of fluid retention. A recent clinical trial of newly diagnosed chronic myelogenous leukemia indicated that nilotinib was superior to imatinib in terms of potency and selectivity of BCR-ABL inhibition; reduction of progression events; absence of a response plateau [14], [15].

Our more recent study revealed an inhibitory effect of nilotinib on proliferation, viability and vitality of PNF-derived Schwann cells and nerve sheath tumor cells *in vitro* with 50% inhibitory concentration (IC_50_) values lower than that of imatinib in our previous study [16], [17]. However, the experimental settings were different in the two *in vitro* studies and an *in vivo* study was not performed. In the present study, we comparatively studied efficacies of the two drugs on PNF *in vitro* on cultured tumor cells and *in vivo* on xenografted tumor fragments in mice.

## Materials and Methods

### Ethical approvals

For the donation of human biological samples approval OB-061/05 by the Institutional Review Board of the University Hospital Hamburg-Eppendorf; for animal studies approval Hamburg 112/11 by the Animal Care and Use Committee of Hamburg.

### Specimen and *in vitro* study

The donor of the study specimen was a 12-year-old female NF1 patient, diagnosed according to the modified National Institutes of Health criteria [18]. A parent of the patient gave informed written consent in addition to assent from the patient. The Institutional Review Board approved the study (OB-061/05). Her PNF was operated at the Department of Maxillofacial Surgery, University Hospital Hamburg-Eppendorf. A part of the tumor was kept in Hanks buffered saline and delivered into the laboratory for cell culture and for xenografting. Schwann cells from the PNF were cultured and identified as previously described [16].

After ensuring purity of 85%, PNF-derived Schwann cells were treated with nilotinib and imatinib (Novartis Pharma AG, Switzerland), each at 0, 5, 10, 15 and 20 µM for 10 days. Cell proliferation and viability assays were performed as previously described [16].

### RNA expression comparison of PNF-segments

To assess variability between PNF grafts, we performed RNA-sequencing from 4 pieces of the original tumor sample and from one unrelated PNF. RNA was isolated from snap-frozen tumors using Trizol. and mRNA was reverse transcribed using SuperScript III First-Strand Synthesis System for RT-PCR (Invitrogen, Carlsabd, CA). Sequencing libraries were prepared using TruSeq Stranded Total RNA Sample Preparation kit (Illumina, San Diego, CA) Libraries were sequenced on a Genome Analyzer IIx (Illumina, San Diego, CA). Alignment and analysis was performed using a Galaxy server and open source Chipster software (http://chipster.csc.fi/) as well as R bioconductor (http://www.bioconductor.org/) tools for calculating similarity clusters on a Galaxy server.

### Xenografting and treatment

The care and use of laboratory animals were carried out in strict accordance with the local animal care and use committee's research council's guide (Approval No.: Hamburg 112/11). Athymic nude mice (female, nu/nu Balb/c, 6 weeks, 20 g) were obtained from Charles River Laboratories (Sulzfeld, Germany).

**Figure 1:**
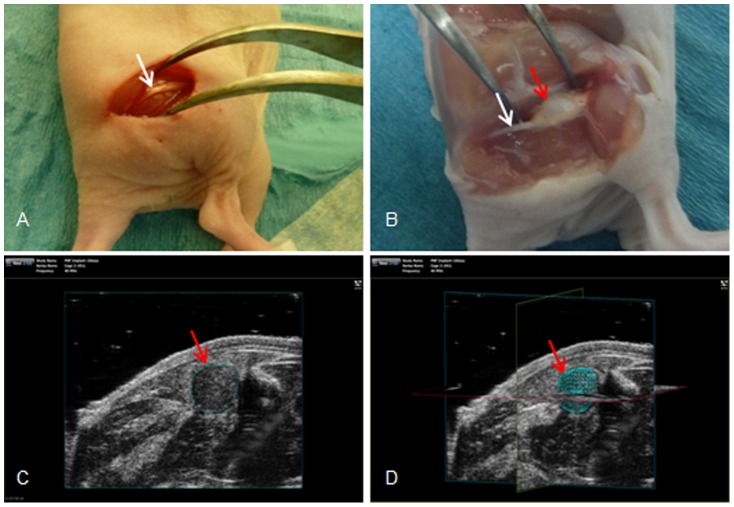
Xenograft on sciatic nerve in mouse. (A) The exposed sciatic nerve (white arrow) for implantation, (B) a PNF xenograft (red arrow) integrated onto the sciatic nerve, (C) images and (D) three-dimensional reconstruction of a xenograft by (red arrow) a Vevo 2100 micro-imaging system.

The xenografting of fragments of PNF was carried out as described previously [17], [19]. Briefly, a small incision was made into the skin to expose the right sciatic nerve and an incision was made into the sciatic nerve, under which one tumor piece was orthtopically implanted (Fig. 1A, B). After confirming successful xenografting by ultrasound scanning one week later, the 23 mice were randomly allocated into three groups: 8 as controls, 8 for nilotinib treatment and 7 for imatinib treatment.

Nilotinib (as the hydrochloride salt) was diluted in 10% N-methyl-pyrrolidinone and 90% polyethylene glycol 300. Imatinib (as the mesylate salt) was dissolved in sterile water. Oral administration at a daily dose of 100 mg/kg of the drugs was started on day 7 after implantation and was continued to day 35. Body weights of the mice were recorded daily and the drug dosage was adjusted accordingly. Food consumption and general condition of the mice were monitored weekly.

At the end of the treatment, animal blood was collected 3 hours after the last oral administration of nilotinib for plasma preparation. Nilotinib concentration was measured using liquid chromatography-tandem mass spectrometry [20].

### Ultrasound monitoring of xenografts

Sonographic measurement was performed using a Vevo 2100 micro-imaging system (VisualSonics, Amsterdam, Netherlands), which is a high-frequency, high-resolution digital imaging platform with linear technology and color Doppler mode. Xenografts were verified after 7 days post transplantation and measured weekly during the whole treatment period of 4 weeks. Three-dimensional images of the xenografts were generated and analyzed using the Vevo software version 5.0.0 to calculate their size in volume (Fig. 1B–D).

### Cytotoxicity of mouse spleen cells

Non-adherent spleen cells were harvested from the mice after erythrocyte- depriving and 3 hours adherence at 37°C. Cytotoxic efficacy of the non-adherent spleen cells was assessed by adding them as effector cells to PNF-derived Schwann cells as target cells at a ratio of 1∶10 for 4 h using the CytoTox 96 Non-Radioactive Cytotoxicity assay (Promega, Fitchburg, WI).

### Statistical analysis

The effect of the drugs *in vitro* was evaluated using Student's *t*-test and the IC_50_ was calculated. Sizes of each xenograft and body weight of the mice were normalized against the corresponding initial values. Time course of size change of the xenografts and body weight increase of the mice in the three groups were compared with each other using a linear mixed model. Pearson correlation coefficients were calculated for the relationships between the reduction of tumor volumes and the cell cytotoxicity, and between the reduction of tumor volumes and the nilotinib concentrations. *P*<0.05 was considered significant. All averaged data were represented as the mean_±standard deviation_.

## Results

### Nilotinib inhibits PNF cells more potently than imatinib

**Figure 2:**
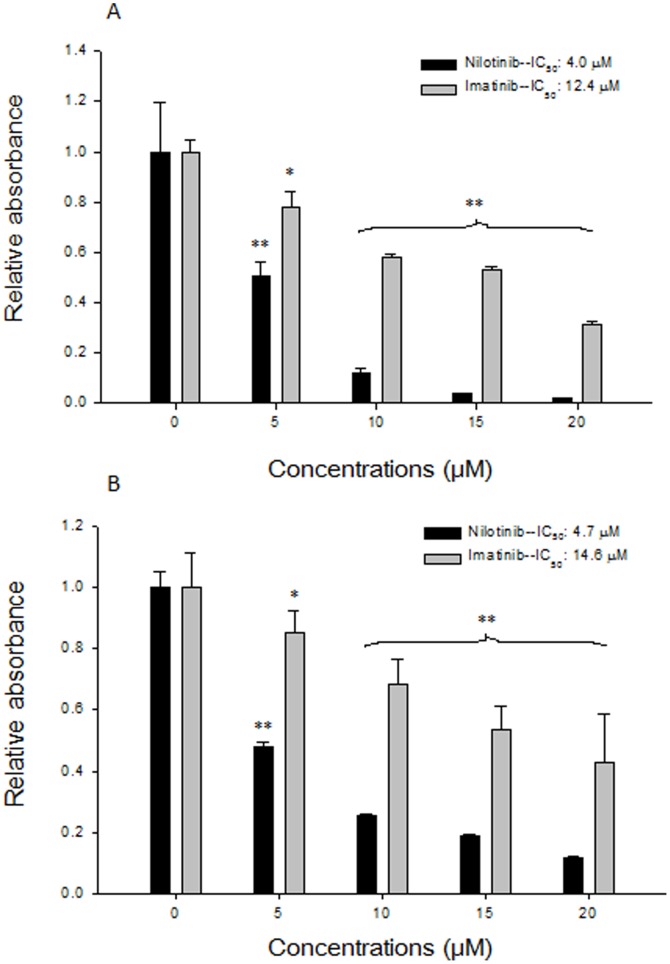
Effects of imatinib and nilotinib on proliferation (A) and viability (B) of PNF-derived Schwann cells. Data are absorbance normalized to that of untreated controls. Significant (*P*<0.05) and highly significant (*P*<0.001) differences were marked with * and **, respectively.

Both nilotinib and imatinib dose-dependently inhibited proliferation and viability of the PNF-derived Schwann cells (Fig. 2). However, the IC_50_ values of nilotinib were 4.0 and 4.7 µM, much lower than the 12.4 and 14.6 µM of imatinib.

### Nilotinib suppressed PNF-xenografts more potently than imatinib

**Figure 3:**
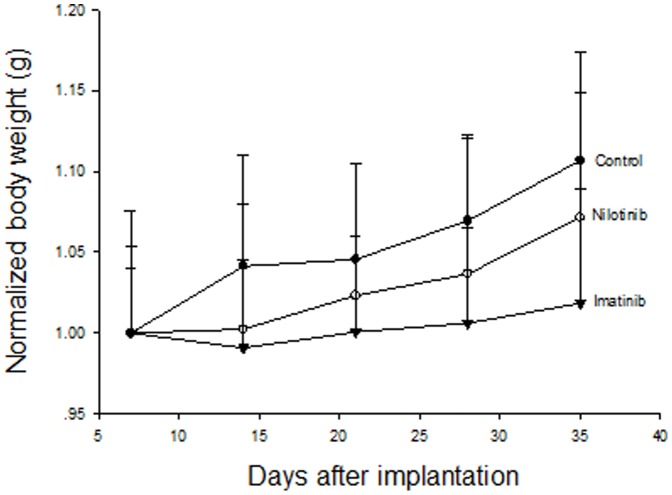
Change of body weights of mice in the three groups over whole experiment period of 35 days. Standard deviations are shown in single direction.

All animals tolerated xenografting and treatments well without visible signs of toxicity and gross abnormalities. General conditions of mice were also compatible among the untreated and the two treated groups. The body weights of mice increased 10% (23.9_±1.0_ to 26.4_±1.6_g) in the control group and 7% (23.4_±1.8_ to 25.1_±1.8_g) in the nilotinib group but not in the imatinib group (2% = 23.3_±1.2_ to 23.7_±1.7_g) over the 28-days of treatment period (Fig. 3). Only the difference between the imatinib group and the control group was significant (P<0.05).

**Figure 4:**
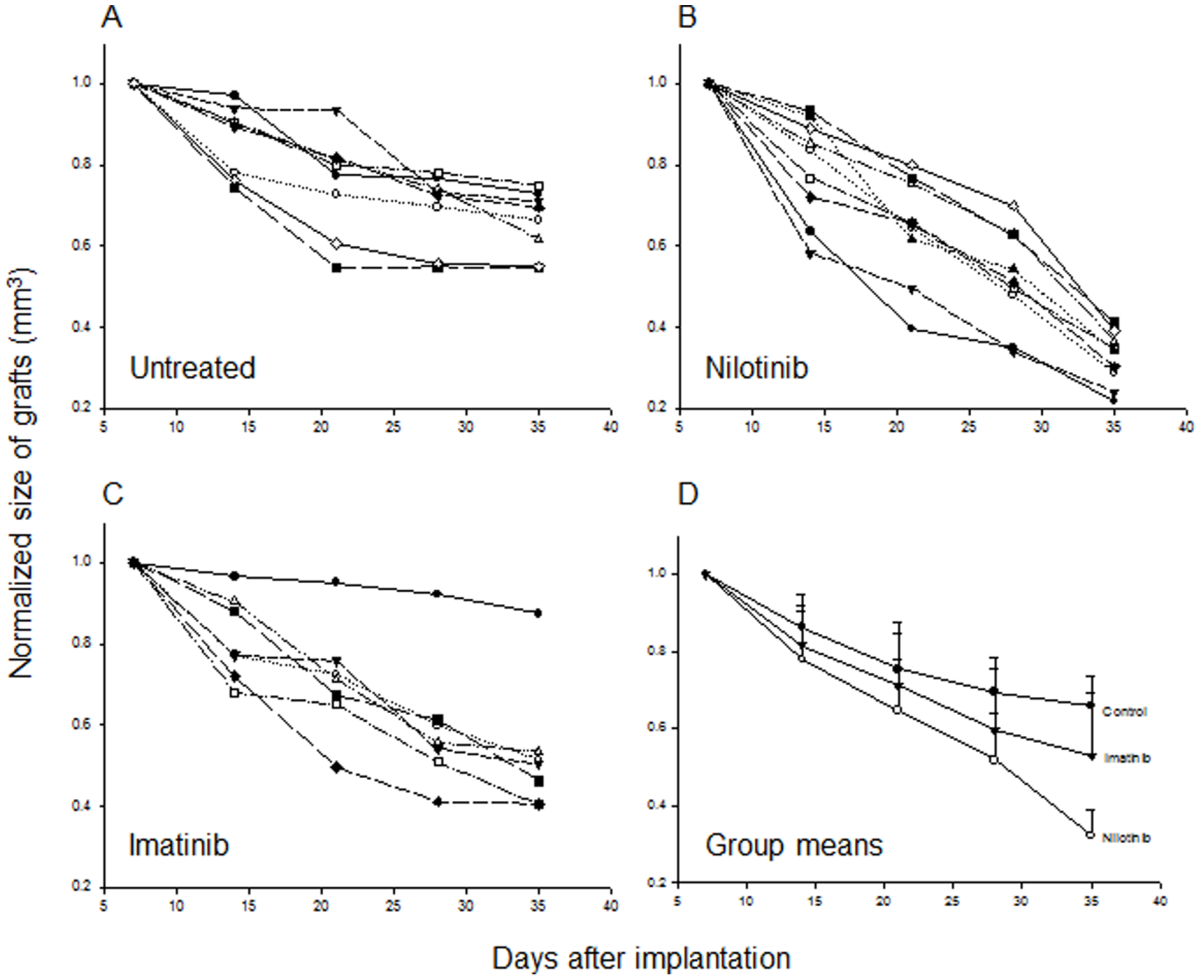
Change of xenograft size in each mouse over the 4-weeks of treatment period in the untreated (A), nilotinib (B) and imatinib (C) groups. Group-means (D) differed significantly among the three groups (P<0.05).

The initial sizes of the xenografts were comparable among the control, nilotinib and imatinib groups, which were 6.0_±3.7_ mm^3^, 5.9_±2.5_ mm^3^ and 5.2_±3.4_ mm^3^, respectively. Grafts decreased in size in all mice for the first two weeks and stabilized and slightly decreased in untreated mice (Fig. 4A). In contrast, size of the xenografts decreased in mice treated with nilotinib (Fig. 4B) or imatinib (Fig. 4C) continued. The decrease in xenograft size was significantly more profound in the nilotinib group than in the untreated group (68_±7_% vs. 33_±8_%, P<0.05) and than in the imatinib group (47_±15_%, P<0.05, Fig. 4D).

Peak plasma nilotinib concentration was 6.6_±1.1_ µM. No correlation was observed between the extent of reduction of tumor volumes and the plasma nilotinib concentrations (r = 0.24, P>0.05). RNA sequencing of 4 different pieces from the original PNF that was used for grafting showed similar but not identical expression patterns, indicating biological variability of the different pieces (data not shown).

### Cytotoxicity of mouse spleen cells

**Figure 5:**
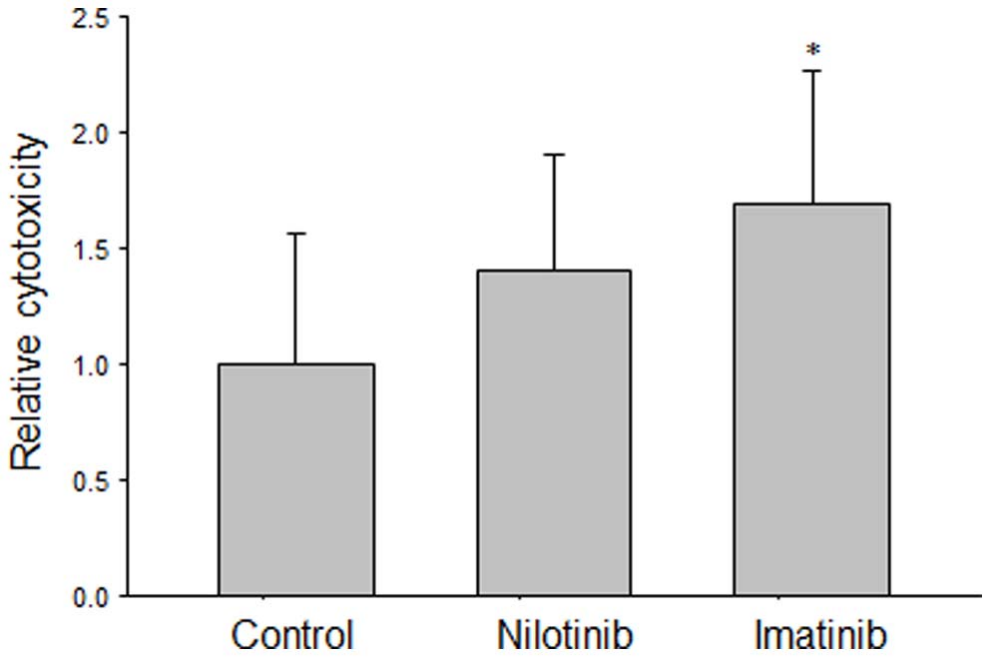
Imatinib significantly (*P<0.05) elevated cytotoxicity of mouse spleen cells on cultured PNF Schwann cells. The elevation by nilotinib was not significant.

Imatinib and nilotinib elevated cytotoxicity of mouse spleen cells on cultured PNF Schwann cells significantly, 21.1_±7.2_% and 17.6_±6.2_%, respectively vs. 12.5_±7.1_% in spleen cells of untreated mice whereas imatinib was significantly more potent than nilotinib (P<0.05, Fig. 5). There was no correlation between the reduction of tumor volumes and the cytotoxcity of mouse spleen cells (r = 0.53, P>0.05).

## Discussion

We showed that nilotinib inhibited proliferation of PNF-derived Schwann cells *in vitro* substantially more potently than imatinib. The applied IC_50_ values of 4.0 µM and 12.4 µM were compatible to the plasma concentrations of the two drugs in patients [12], [21], legitimating their potential clinical application for PNF.

No growth was observed in any of the xenografts, in concordance with the result of the natural history study of PNF, which showed no growth in the majority of cases and very slow growth over years in the few cases where the tumors do grow. It is therefore unreasonable to expect any detectable growth of small xenografts in short experiment periods of less than 2 months. In fact, xenografts tend to shrink in the 2 to 3 weeks after the implantation, likely due to clearance of pre-grafting and post-grafting cell death. Drug effect can therefore be described as an increase in graft size reduction, which is more profound after 2 to 3 weeks. Indeed, we could detect significantly more grafts size reduction in the drug-treated mice than in the untreated control mice. Furthermore, the significantly more potent effect of nilotinib over imatinib could also be demonstrated in this *in vivo* model. This is of high relevance for patients with PNF, which suffer mostly from the secondary tumor size effects.

Recently, a growth deceleration of PNF was reported in children treated with imatinib [22]. In concordance, we also observed a deceleration of body weight increase in mice treated with imatinib. Judging from body weight, nilotinib was better tolerated than imatinib by the mice, indicating a better side effect profile of the former.

Since off-target effects of imatinib have been reported, among them the effect of activating natural killer cells [23], we measured cytotoxicity of spleen cells of the treated mice. We found elevated cytotoxicity of spleen cells in imatinib -treated mice and to a lesser extent, in nilotinib-treated mice. This finding suggests that imatinib might have an immune-activating component in its anti-PNF effect, such that its pharmacological mechanism differs from that of nilotinib.

In summary, our data reveal a more potent antitumor effect of nilotinib on PNF than imatinib *in vitro* and *in vivo*, suggesting the potential clinical application of nilotinib for PNFs.
